# Germline *BRCA1/2* Mutations in a Large Clinic-Based Cohort of Patients with Metastatic Breast Cancer in France

**DOI:** 10.3390/cancers18050851

**Published:** 2026-03-06

**Authors:** Guillaume Meynard, Victor Pereira, Sophie Paget-Bailly, Elodie Klajer, Laura Mansi, Loïc Chaigneau, Nathalie Meneveau, Marie Justine Paillard, Fernando Bazan, Erion Dobi, Cristian Villanueva, Zohair Selmani, Julien Viot, Lorraine Dalens, Morgan Goujon, Marie-Agnès Collonge-Rame, Céline Populaire, Aurélia Meurisse, Xavier Pivot, Elsa Curtit

**Affiliations:** 1 Department of Medical Oncology, Jean-Minjoz University Hospital Centre, F-25000 Besançon, France; gmeynard@chu-besancon.fr (G.M.); lmansi@chu-besancon.fr (L.M.); lchaigneau@chu-besancon.fr (L.C.); nmeneveau@chu-besancon.fr (N.M.); mjpaillard@chu-besancon.fr (M.J.P.); ferbzn@hotmail.com (F.B.); e1dobi@chu-besancon.fr (E.D.); zselmani@chu-besancon.fr (Z.S.); jviot@chu-besancon.fr (J.V.); ldalens@chu-besancon.fr (L.D.); m1goujon@chu-besancon.fr (M.G.); elsa.curtit@gmail.com (E.C.); 2 Marie et Louis Pasteur University, EFS, INSERM, UMR RIGHT, F-25000 Besançon, France; spaget@chu-besancon.fr (S.P.-B.); ahusse@chu-besancon.fr (A.M.); 3 Department of Methodology and Quality of Life in Oncology, Jean Minjoz University Centre, F-25000 Besançon, France; 4 Montpellier Cancer Centre, Clémentville Clinic, Medical Oncology, 34070 Montpellier, France; cvillanueva@oncoclem.org; 5 Department of Oncobiology, Jean Minjoz University Hospital, 25000 Besançon, France; ma1collongerame@chu-besancon.fr (M.-A.C.-R.); cpopulaire@chu-besancon.fr (C.P.); 6 Department of Medical Oncology, ICANS, 67200 Strasbourg, France; x.pivot@icans.eu

**Keywords:** *BRCA*, genetic screening, metastatic breast cancer, breast cancer susceptibility gene, prevalence

## Abstract

Objectives: This study aimed to fill the data gap regarding the prevalence of germline (g) *BRCA1/2* mutations in patients with metastatic breast cancer (mBC) in France. Methods: A prospective g*BRCA1/BRCA2* mutation analysis was proposed to all patients with mBC treated in seven French centers between 19 February and 30 November 2015. The BRCA TrueTM test (Pathway Genomics^®^, San Diego, CA, USA) analyzed the coding and flanking regions of *BRCA1* and *BRCA2* genes using next-generation sequencing, Sanger sequencing, and multiplex ligation-dependent probe amplification. Results: Among 407 included mBC patients, 11 (2.7%) carried pathogenic g*BRCA1/2* mutations. Of these, five (45%) would not have met standard criteria for genetic screening. Compared with non-carriers, g*BRCA1/2* carriers were significantly younger at mBC diagnosis (47.5 vs. 60.7 years, *p* = 0.0006), had higher-grade tumor histology (*p* = 0.044), and had a higher rate of contralateral recurrence (36.4% vs. 11.6%, *p* = 0.035), with comparable adjusted survival (median overall survival 74.9 vs. 100.1 months, *p* = 0.97). Variants of uncertain significance were identified in 17 (4.2%) patients. Conclusions: The 2.7% prevalence of g*BRCA1/2* mutations in this prospective French mBC cohort was relatively low. Nearly half of the mutation carriers would not have been routinely referred for oncogenetic counseling, underscoring the potential value of broader genetic screening in this population.

## Simple Summary

Inherited mutations in the *BRCA1* and *BRCA2* genes are well known in early breast cancer, but much less is known about how often they occur in patients with metastatic breast cancer, especially in routine clinical practice. In this study, we offered genetic testing to a large group of patients with metastatic breast cancer treated in several hospitals in eastern France, regardless of their age or family history. We found that a small but clinically important proportion of patients carried a *BRCA1* or *BRCA2* mutation, and nearly half of them would not have been identified using standard referral criteria. These results suggest that broader access to genetic testing could help better identify patients who may benefit from targeted treatments, such as PARP inhibitors, and support more equitable implementation of precision medicine in metastatic breast cancer.

## 1. Introduction

Genetic susceptibility to breast cancer is linked to several genes associated with well-established hereditary cancer syndromes, including the *BRCA1* and *BRCA2* tumor suppressor genes [1,2]. Germline mutations in *BRCA1* and *BRCA2* account for approximately 5–10% of familial breast cancer cases [3]. Women carrying *BRCA1* or *BRCA2* mutations face an estimated lifetime breast cancer risk ranging from 50% to 80% [4,5].

The reported prevalence of germline *BRCA1/2* (g*BRCA1/2*) mutations varies between 1.7 and 33.3% across different populations of patients with breast cancer [6,7,8,9,10]. Higher g*BRCA1/2* mutation rates have been reported in patients with a strong family history of breast cancer [6,7,11], triple-negative disease (i.e., estrogen-receptor-negative, progesterone-receptor-negative, and human epidermal growth factor receptor type 2 [HER2]-negative) [7,8,12], younger age at diagnosis [6,7,8,11,12], and Ashkenazi Jewish ancestry [6,11]. The prevalence of g*BRCA1/2* mutations in metastatic breast cancer (mBC) patients has been reported to be 21% in a US-based single-center study [13] and 9.7% in an international multicenter study [14]. However, to date, the prevalence of g*BRCA1/2* mutations in mBC patients in France remains unknown. Identifying g*BRCA1/2* mutations in this population has significant implications, particularly for treatment with poly (ADP–ribose) polymerase (PARP) inhibitors.

*BRCA1* and *BRCA2* genes encode proteins involved in the repair of DNA double-strand breaks through the homologous recombination repair pathway. Genomic aberrations disrupting DNA repair mechanisms have been associated with sensitivity to platinum-based chemotherapy [15,16] and to poly (ADP–ribose) polymerase (PARP) inhibitor therapy [17,18,19]. PARP inhibitors, such as olaparib and talazoparib, are approved for the treatment of HER2-negative mBC patients with g*BRCA1/2* mutations based on OlympiAD and EMBRACA trials, respectively [20,21,22]. According to the guidelines from the European Society of Medical Oncology (ESMO), the American Society of Clinical Oncology (ASCO), and the National Comprehensive Cancer Network (NCCN), patients with pathogenic g*BRCA1/2* mutations and HER-2-negative mBC should be considered for PARP inhibitor treatment, regardless of their hormone receptor status, as an alternative to chemotherapy [23,24]. This present study aimed to determine the frequency of g*BRCA1/2* mutations using full-sequence analysis and multiplex ligation-dependent probe amplification (MLPA) in a large, unselected, clinic-based cohort of patients with mBC.

## 2. Materials and Methods

### 2.1. Study Design

This was a prospective, multicenter cohort study conducted in seven centers located in the Franche-Comté region of France, which has a population of over 1.1 million people.

### 2.2. Patients and Procedures

Patients eligible for inclusion in the study had histologically confirmed mBC and unknown g*BRCA1/2* mutational status; thus, no pre-study sample size calculation was performed. Study participants were enrolled between 19 February and 30 November 2015 and followed as part of routine care at each site until death, withdrawal of consent, or for up to five years. All participants provided written informed consent. According to French legislation applicable at the time of the study, this non-interventional research did not require approval from a Comité de Protection des Personnes (CPP). The study was conducted in accordance with the ethical principles of the Declaration of Helsinki and applicable institutional requirements.

Participants provided a blood sample for germline *BRCA1/2* DNA analysis. Patient demographics, family history of cancer, and treatment data were collected using an electronic case report form.

### 2.3. BRCA1 and BRCA2 Testing

Germline DNA (gDNA) was extracted from peripheral blood samples and analyzed by next-generation sequencing (NGS) and Sanger sequencing at the Pathway Genomics Laboratories (San Diego, CA, USA) using the BRCA True^TM^ test (Pathway Genomics Laboratories, San Diego, CA, USA). The test targeted the coding and flanking regions of the *BRCA1* and *BRCA2* genes. The gDNA was first assessed for quality and quantity, then enriched for the targeted exons and flanking regions using polymerase chain reaction (PCR) with specific primers. NGS was performed on the enriched DNA to detect variants. Sanger sequencing was used for regions insufficiently covered by NGS and to confirm suspected pathogenic or novel variants.

Large rearrangements, deletions, and duplications in *BRCA1/2*, which are missed by direct sequencing [25], were identified via MLPA.

Results were classified according to the American College of Medical Genetics (ACMG) guidelines as pathogenic mutation or variants of uncertain significance (VUS). Patients with pathogenic mutations or VUS were referred for genetic counseling. Patients were classified into two groups: *BRCA1/2*-positive (those with a pathogenic g*BRCA 1/2* mutation) or *BRCA1/2*-negative (those without a pathogenic g*BRCA1/2* mutation or with VUS).

### 2.4. Study Outcomes

Baseline demographics (age at diagnosis of primary breast cancer and of mBC) and disease characteristics (histological grade, *HER-2* and hormone-receptor status, the presence of breast cancer in the contralateral breast, and the presence of metachronous ovarian cancer) were evaluated in both groups, *BRCA1/2*-positive and *BRCA1/2*-negative groups. Overall survival (OS), defined as the time from mBC diagnosis until death from any cause, was also analyzed. Patients alive were censored at the date of their last follow-up.

### 2.5. Statistical Analysis

The primary goal of the study was to assess the prevalence of g*BRCA1/2* mutations among patients with unknown g*BRCA1/2* status in the local mBC population. Comparisons between *BRCA1/2*-positive and *BRCA1/2*-negative groups were descriptive due to the non-randomized design. Because of the small sample size across strata, we used Fisher’s exact test to compare the distribution of selected categorical variables between the two groups. The Kruskal–Wallis test was used to compare continuous variables. The Kaplan–Meier method was employed to estimate median OS with 95% confidence intervals (CIs). The log-rank test was used to compare survival between groups. The reverse Kaplan–Meier method was used to estimate median follow-up time. Given the limited number of g*BRCA1/2* mutation carriers, all comparative and survival analyses were considered exploratory and hypothesis-generating. No formal sample size calculation was performed, and no multivariable modeling was conducted due to limited statistical power.

## 3. Results

### 3.1. gBRCA1/2 Status in the mBC Population

**Figure 1:**
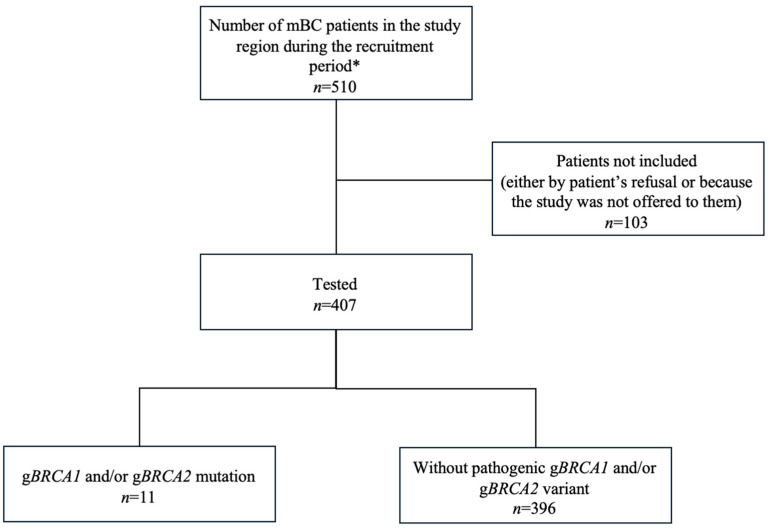
Population flowchart.

**Figure 2:**
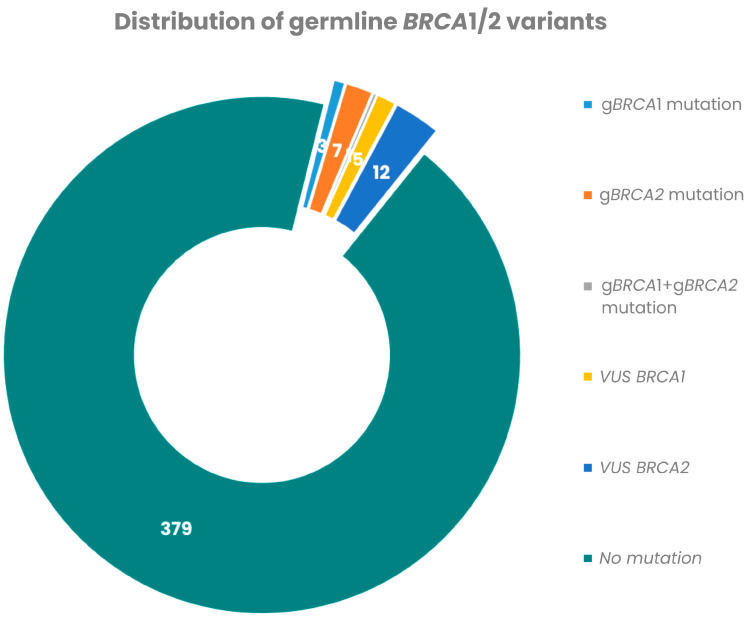
gBRC1/2 variants in unselected patients with mBC.

A total of 510 mBC patients from the seven participating hospitals were potentially eligible for participation in the study, based on the number of patients with mBC whose records were available in the regional health database. Of these, 407 patients underwent testing of g*BRCA1*/*BRCA2* mutations, and the mutational status was successfully determined for all. Pathogenic g*BRCA1/2* mutations were identified in 11/407 patients (2.7%) (Figure 1). *BRCA2*-only (*n* = 7) mutations were the most common, affecting 7/407 patients (1.7% of the cohort, 63.6% of g*BRCA1/2* carriers). Three patients were *BRCA1-only* mutation carriers (0.7% of the cohort, 27.3% among g*BRCA1/2* carriers), while one patient had both *BRCA1* and *BRCA2* germline mutations (Figure 2). Each mutation carrier harbored a unique pathogenic variant (Supplementary Table S1). VUS were found in 17/407 patients (4.2%) (Figure 2).

### 3.2. Patients and Tumor Characteristics According to BRCA1/2 Status

Compared to non-carriers, g*BRCA1/2* carriers were significantly younger at diagnosis of primary breast cancer (median age 56.6 years versus 45.0 years, respectively, *p* = 0.0029) and at diagnosis of mBC (median age 60.7 years versus 47.5 years, respectively, *p* = 0.0006) (Table 1). There were several significant clinical and pathological differences between *BRCA1/2*-positive and *BRCA1/2*-negative patients. g*BRCA1/2* mutations were significantly associated with a high histological grade (*p* = 0.044) compared to non-carriers. All g*BRCA1/2* mutation carriers had HER2-negative breast cancer. All patients with a g*BRCA2* mutation (*n* = 7) presented with hormone receptor-positive mBC, whereas all patients with a g*BRCA1* mutation (*n* = 4) had triple-negative mBC. The prevalence of *gBRCA* gene mutations among the metastatic HER2-negative breast cancer patient population was 3.0% (11/368). Contralateral breast tumors occurred more frequently in 4/11 (36.4%) patients with g*BRCA1/2* mutation and 46/396 (11.6%) patients without g*BRCA1/2* mutation (*p* = 0.035) (Table 2). None of the patients in the g*BRCA1/2*-positive or g*BRCA1/2*-negative groups developed metachronous ovarian cancer.

### 3.3. Universal BRCA1/2 Mutation Screening

Universal *BRCA1/2* mutation screening in this mBC patient population detected 11 g*BRCA1/2* mutations. In 2015, the Manchester score (based on personal and family history) was widely applied in oncogenetics to help make decisions about performing oncogenetic research. A family or individual score ≥ 16 is a “strong indication for genetic research,” corresponding to a g*BRCA1/2* mutation probability of 10%. Based on the Manchester score, only 6/11 patients with a g*BRCA1/2* mutation identified in our study would have been assessed for genetic predisposition to breast cancer. Universal screening, irrespective of the Manchester score, allowed detection of g*BRCA1/2* mutations in five additional patients.

### 3.4. Survival Data

**Figure 3:**
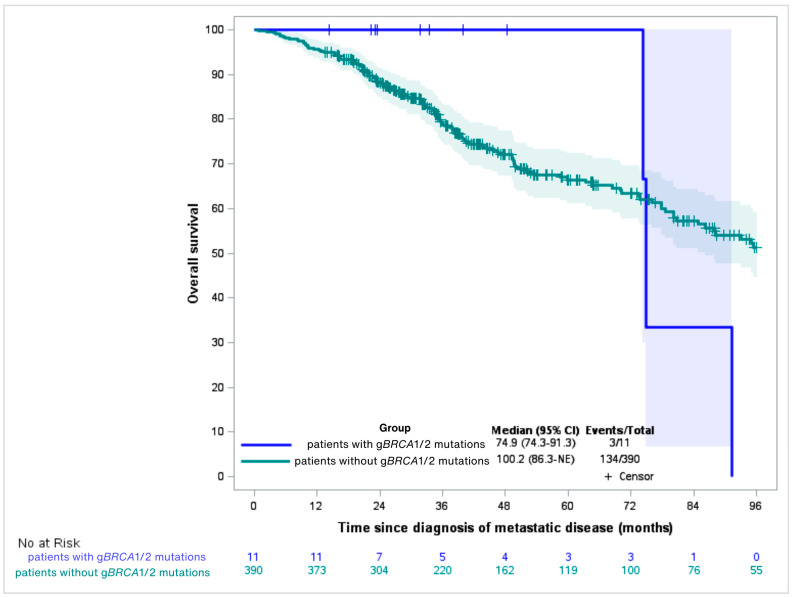
Overall survival from diagnosis of metastatic disease, by gBRCA1/2 mutational status.

Median follow-up was 53.3 months (95% CI: 47.64–65.54); eight patients (2%) were lost to follow-up. Median OS was 74.9 months in g*BRCA1/2*-positive (95% CI: 74.3–91.3) and 100.1 months in g*BRCA1/2*-negative patients (95% CI: 84.9–170.4), with no statistically significant difference between the two groups (logRank = 0.97) (Figure 3).

## 4. Discussion

This was the first prospective study to assess the prevalence of germline *BRCA1/2* (g*BRCA1*/*2)* mutations in a large, clinic-based cohort of unselected patients with mBC in France. Full sequencing of the *BRCA1/2* genes was performed using next-generation sequencing (NGS), supplemented by Sanger sequencing and multiplex ligation-dependent probe amplification (MLPA). We identified that pathogenic g*BRCA1/2* mutations were found in 2.7% of patients, a considerably lower prevalence compared to international and U.S. mBC cohorts, where g*BRCA1/2* mutation prevalence was reported at 9.7% and 21%, respectively [13,14]. Notably, none of the patients with HER2-positive tumors in our cohort carried a g*BRCA* mutation. Among those with HER2-negative mBC, the prevalence of g*BRCA* mutations was 3% (11/368). Importantly, about half the mutation carriers would not have routinely been referred for oncogenetic counseling.

Several clinical observations in our cohort are consistent with previous reports. g*BRCA1/2* carriers were younger at diagnosis and experienced more contralateral events, findings that align with other studies [6,7,8,11,12,26,27,28]. We also found an association between g*BRCA1/2* mutations and higher histological tumor grade, consistent with the international BREAKOUT study, which reported a higher incidence of poorly differentiated tumors in g*BRCA1/2* mutation carriers [14]. Additionally, our results support the established link between *BRCA1* mutations and triple-negative breast cancer with a basal-like profile [29,30,31] as well as the association of *BRCA2* mutations with estrogen receptor-positive, HER2-negative tumors exhibiting a luminal profile [30,32].

One patient in our cohort presented with the rare occurrence of double heterozygosity for *BRCA1* and *BRCA2* pathogenic variants, a mutation pattern that the same author group had previously published as a case study. This rare co-occurrence is particularly uncommon in non-Ashkenazi individuals [33]. In the international BREAKOUT study, 1.5% of patients had mutations in both *BRCA1* and *BRCA2* [14], compared to 0.3% in our cohort. The ethnicity of the patients with double heterozygosity in the BREAKOUT study was not reported. Notably, no such cases were identified in a smaller North American mBC cohort [13].

Universal g*BRCA1/2* sequencing in patients with mBC is crucial for optimizing care for both the patient and their family. In 2015, our genetic counseling services used the Manchester Scoring System to determine eligibility for *BRCA1/2* testing [34]. In our cohort, 5 of 11 g*BRCA1/2* mutation carriers would not have met these criteria, suggesting that universal screening could detect 45% more g*BRCA1/2* mutations. However, the relationship between gBRCA1/2 mutations and prognosis remains complex and likely influenced by tumor subtype and treatment exposure. In our study, no survival difference was observed between carriers and non-carriers. These findings must be interpreted cautiously, as no multivariable analyses were performed and important confounders—including tumor subtype, number of treatment lines, platinum exposure, and PARP inhibitor use—were not adjusted for. Recent large cohort analyses have suggested that BRCA mutation carriers, particularly younger patients, may experience worse outcomes depending on clinical context [35]. Therefore, our results should be considered exploratory and hypothesis-generating rather than definitive. In our study, survival outcomes were similar between mutation carriers and non-carriers, in contrast with the findings by Bayraktar et al., who reported worse outcomes for g*BRCA1* mutation carriers compared to g*BRCA2* mutation carriers and non-carriers [13]. However, Maillez et al. recently emphasized that the prognostic impact of g*BRCA1/2* status is likely influenced by tumor subtype (e.g., triple-negative, HER2 status) rather than mutation status alone [36].

Tumors associated with *BRCA1/2* mutations have been shown to be more sensitive to PARP inhibitors. Both olaparib and talazoparib are approved in the European Union, including France, for treating patients with g*BRCA1/2* mutations and HER-2-negative mBC. The OlympiAD trial demonstrated that olaparib significantly improved progression-free survival compared to chemotherapy in these patients [21,22]. Similarly, talazoparib showed significant benefits in the EMBRACA trial [20]. A recent meta-analysis of 43 interventional studies found that PARP inhibitors achieved an objective response rate of 57% and a clinical benefit rate of 73% among mostly germline *BRCA1/2* mutation carriers with mBC, with no significant differences between *BRCA1* and *BRCA2* mutation carriers [37]. According to current international guidelines (2024–2025 updates from ESMO, ASCO, and NCCN), germline *BRCA1/2* testing is recommended for patients with HER2-negative metastatic breast cancer in order to guide therapeutic decision-making, particularly regarding PARP inhibitor eligibility [23,24,38]. Our findings, showing that nearly half of mutation carriers would not have been identified using historical referral criteria, support the shift toward broader and more systematic genetic testing in this setting. Despite these advances, g*BRCA1/2* testing remains underutilized [39].

This study has several limitations. First, patient inclusion was conducted in 2015 and therefore reflects genetic testing practices and therapeutic standards of that period. Since then, access to multigene panel testing has expanded, referral criteria have evolved, and PARP inhibitors have become integrated into routine clinical practice. Consequently, the present prevalence estimates may not fully reflect current testing strategies in metastatic breast cancer. In addition, the relatively small number of mutation carriers limits the strength of subgroup and survival analyses. Another important limitation concerns the absence of detailed treatment exposure data, particularly regarding platinum-based chemotherapy. As the primary objective of this study was to determine the prevalence of germline *BRCA1/2* mutations, systemic treatment variables were not prospectively collected in a standardized manner. In addition, patient inclusion occurred prior to the approval of PARP inhibitors in metastatic breast cancer, and BRCA status did not influence therapeutic decision-making at that time. Therefore, survival comparisons between mutation carriers and non-carriers could not be adjusted for treatment exposure and should be interpreted cautiously as exploratory and hypothesis-generating.

Breast cancer genetic susceptibility may also be linked to mutations in genes beyond *BRCA1/2*, some of which are involved in known hereditary cancer syndromes, such as *TP53*, *PTEN*, *CDH1*, and *PALB2* [40]. The role of genes like *ATM*, *CHEK2*, and *RAD51* in breast cancer risk is still being investigated [41,42]. While our study did not assess mutations in these genes, the BREAKOUT study reported that mutations in *ATM*, *CHEK2*, and *RAD51* were found in less than 3.1% of patients [14]. Importantly, expanding genetic testing to include additional genes may increase the detection of variants of uncertain significance (VUS). Most VUS do not confer a high cancer risk; however, misinterpretation could lead to inappropriate management of the patient and their relatives. Patients with VUS identified in the *BRCA* genes should be referred to specialized genetic counseling services. Further research is needed to clarify the importance of germline gene panel sequencing, somatic sequencing, and clinical VUS interpretation.

## 5. Conclusions

In summary, this study is the first to assess the prevalence of g*BRCA1/2* mutations in a large, prospective, clinic-based cohort of unselected patients with mBC in France. The overall mutation rate of 2.7% reflects the real-world prevalence of g*BRCA1/2* mutations in French mBC patients, which is lower than reported in other countries. Nearly half of the mutation carriers in our cohort would not have been routinely referred for oncogenetic counseling. The 3% prevalence of g*BRCA1/2* gene mutations in HER2-negative mBC patients highlights a subset of individuals who may benefit from treatment with PARP inhibitors.

## Figures and Tables

**Table 1:** Patients and tumour characteristics considering g*BRCA1/2* mutational status.

	No Mutation n = 396		g*BRCA1/2* Mutation n = 11			Total n = 407	
Characteristics	n	%	n	%	*p* Value	n	%
Median age at diagnosis of early breast cancer, y Median	56.6		45		0.0029 *	56.3	
Median age at diagnosis of metastatic breast cancer, y	60.7		47		0.0006 *	60.3	
T stage					0.5705		
- T0	2	0.6	0	0		2	0.5
- T1	74	21	2	20		76	21
- T2	132	37.6	2	20		134	37.1
- T3	81	23.1	3	30		84	23.3
- T4	62	17.7	3	30		65	18
- Missing	45		1			46	
N stage					0.7729		
- N0	104	29.9	3	30		107	29.9
- N1	163	46.8	5	50		168	46.9
- N2	45	12.9	2	20		47	13.1
- N3	36	10.3	0	0		36	10.1
- Missing	48		1			49	
M status					1		
- Primary	176	45.4	5	44.4		181	44.5
- Secondary	220	54.6	6	55.6		226	55.5
Histologic grade					0.0158 *		
- 1	40	11.7	0	0		40	11.4
- 2	211	62.1	3	30		214	61.1
- 3	89	26.2	7	70		96	27.4
- Missing	56		1			57	
Hormonal receptor status					0.3887		
- Positive	333	84.7	8	72.7		341	84.4
- Negative	60	15.3	3	27.3		63	15.6
- Missing	2					3	
Triple negative					0.0135 *		
- Yes	34	8.6	4	36.4		38	9.4
- No	360	91.4	7	63.6		367	90.6
- Missing	2					2	
HER2 status					0.6102		
- Positive	39	9.9	0	0		39	9.6
- Negative	355	90.1	11	100		366	90.4
- Missing	2					2	

g*BRCA1/2*: presence of germline mutation of *BRCA1* or *BRCA2*; HER2: Human Epidermal Receptor growth factor type 2; M: metastasis; N: nodal; n: number; T: tumour; y: year. *: Significant value.

**Table 2:** Personal and familial histories considering BRCA mutational status.

	No Mutation n = 396		g*BRCA1/2* Mutation n = 11			Total n = 407	
Characteristics	n	%	n	%	*p* Value	n	%
Personal history of cancer					0.4162		
No	317	82.8	8	72.7		325	82.5
Yes	66	17.2	3	27.3		69	17.5
Unknown	13					13	
Contralateral cancer					0.035 *		
No	349	88.4	7	63.6		356	87.7
Yes	46	11.6	4	36.4		50	12.3
Unknown	1		0			1	
Familial history of breast cancer					0.0022 *		
No	283	80.4	4	36.4		287	79.1
Yes	69	19.6	7	63.6		76	20.9
Unknown	44		0			44	
Familial history of ovarian cancer					0.0161 *		
No	348	98.6	9	81.8		357	98.1
Yes	5	1.4	2	18.2		7	1.9
Unknown	43		0			42	
Familial history of other cancer					0.0129 *		
No	284	80.3	5	45.5		289	79.2
Yes	70	19.7	6	54.5		76	20.8
Unknown	42		0			42	

g*BRCA1/2*: presence of germline mutation of *BRCA1* or *BRCA2*. *: Significant value.

## Data Availability

Anonymized data analyzed during the current study are available from the corresponding author on reasonable request.

## Supplementary Materials

The following supporting information can be downloaded at https://www.mdpi.com/article/10.3390/cancers18050851/s1. Table S1: Listing of germline mutation of *BRCA1* and *BRCA2*.

## Abbreviations

The following abbreviations are used in this manuscript:

ACMG	American College of Medical Genetics
ASCO	American Society of Clinical Oncology
CI	Confidence interval
ESMO	European Society of Medical Oncology
g	Germline
HER2	Human epidermal growth factor receptor type 2
mBC	Metastatic breast cancer
MLPA	Multiplex ligation-dependent probe amplification
NCCN	National Comprehensive Cancer Network
NGS	Next generation sequencing
OS	Overall survival
PARP	Poly (ADP–ribose) polymerase
PCR	Polymerase chain reaction
VUS	Variant of uncertain significance
