# Supplementary material for: Germline BRCA1/2 Mutations in a Large Clinic-Based Cohort of Patients with Metastatic Breast Cancer in France
Source: Cancers (Basel). 2026 Mar 6;18(5):851. doi: 10.3390/cancers18050851 (PMC12984906; doi:10.3390/cancers18050851)
Supplement: Supplementary file 1 [file cancers-18-00851-s001.zip › cancers-4139484-supplementary.pdf]

Supplementary Material

Supplementary Table S1: Listing of germline mutation of *BRCA1* and *BRCA2*.

Patient	Age	Gene	Pathogenic variant	Variant type	Molecular consequence
B17	46	g*BRCA2*	c.3599_3600delGT	Deletion	Frameshift
B52	46	g*BRCA2*	c.4889C>G	Single nucleotide variant	Nonsense
B55	40	g*BRCA2*	c.3680_3681delTG	Deletion	Frameshift
B73	46	g*BRCA1*	c.1016dupA	Duplication	Frameshift
		g*BRCA2*	c.6814delA	Deletion	Frameshift
B94	46	g*BRCA2*	c.8167G>C	Single nucleotide variant	Missense
B228	29	g*BRCA2*	c.9883C>T	Single nucleotide variant	Nonsense
B262	44	g*BRCA2*	c.1796_1800delCTTAT	Deletion	Frameshift
B272	54	g*BRCA1*	c.798_799delTT	Deletion	Frameshift
B290	61	g*BRCA1*	c.171delG	Deletion	Frameshift
B401	40	g*BRCA2*	c.5835_5842dupATCACCTT	Duplication	Frameshift
B418	42	g*BRCA1*	c.191G>A	Single nucleotide variant	Missense
